# Evaluation of the impact of S-adenosylmethionine-dependent methyltransferase inhibitor, 3-deazaneplanocin A, on tissue injury and cognitive function in mice

**DOI:** 10.18632/oncotarget.25062

**Published:** 2018-04-17

**Authors:** Eva Lhuissier, Juliette Aury-Landas, Valentine Bouet, Céline Bazille, Yohann Repesse, Thomas Freret, Karim Boumédiene, Catherine Baugé

**Affiliations:** ^1^ Normandie Univ, UNICAEN, BioConnecT, Caen, France; ^2^ Normandie Univ, UNICAEN, INSERM, COMETE, Caen, France; ^3^ CHU de Caen, Service d’Anatomie Pathologie, Caen, France; ^4^ Normandie Univ, UNICAEN, INSERM, EFS, PhIND, Caen, France; ^5^ CHU de Caen, Hématologie biologique, Caen, France; ^6^ Normandie Univ, UNICAEN, CURB-BRP, Caen, France

**Keywords:** cognitive functions, antitumoral drug, epigenetic, toxicity

## Abstract

Cancer patients display cognitive impairment due, at least partly, to the treatments. Additionally, chemotherapeutic treatments can lead to organ injury, limiting their use, and are likely to have negative impacts on patients’ quality of life. The aim of this study was to investigate the toxicity of 3-Deazaneplanocin A (DZNep) on several tissues and organs, as well as on cognitive functions. DZNep is an inhibitor of S-adenosylmethionine-dependent methyltransferase (in particular of the histone methyltransferase EZH2) which showed antitumoral functions in preclinical trials but whose effects on behavior and on organs (side effects) are not known.

Chronic injections of DZNep were performed intraperitoneally in male NMRI mice (2 mg/kg; i.p.; three times per week) during 8 weeks. A follow-up of body weight was assessed during all experiments. Histological analysis were performed on several organs. EZH2 expression and H3K27me3 were assayed by western-blot. Several behavioral tests were performed during treatment and 2 weeks after. A particular focus was made on spontaneous locomotor activity, cognitive functions (spontaneous alternation and recognition memory), and anxiety- and depression-related behavior. Hematological modifications were also assessed.

Chronic DZNep treatment transiently reduced animal growth. It had no effect on most organs but provoked a reversible splenomegaly, and persistent testis reduction and erythropoiesis. DZNep administration did not alter animal behavior.

In conclusion, this study is encouraging for the use of DZNep for cancer treatment. Indeed, it has no effect on animal behavior, conferring an advantageous safety, and induces irreversible side effects limited on testis which are unfortunately found in most chemotherapy treatments.

## INTRODUCTION

Cancer treatments, including chemotherapy, may induce side effects on the bone marrow, heart, cardiac, digestive system or testis, and often cause nausea, alopecia, or even cognitive impairments [1]. These cognitive impairments include alteration of concentration, memory, executive functions, and processing speed, and are often named “chemofog” or “chemobrain” [2, 3]. These cognitive disorders as well as tissue injuries have major consequences on patient quality of life, return to work, or autonomy.

During the last decade, new antitumoral drugs have been developed. The understanding of epigenetic alterations, such as histone methylation, enabled especially emerging targets for cancer therapy. Inhibitors of histone methylation are currently in development. 3-deazaneplanocin A (DZNep), a cyclopentanyl analog of 3-deazaadenosine, inhibits the activity of S-adenosylhomocysteine hydrolase (SAHH), leading to cellular accumulation of S-adenosylhomocysteine (SAH), which in turn, represses the S-adenosyl-methionine-dependent histone lysine methyltransferase activities [4]. In particular, DZNep inhibits the Polycomb Repressive Complex 2 (PRC2), and particularly its catalytic subunit Enhancer of Zeste Homolog 2 (EZH2), inhibiting the trimethylation of the lysine 27 on histone H3 (H3K27me3) [5].

Initially investigated for its antiviral properties, DZNep has also demonstrated antitumoral activity [6]. It induces apoptosis, inhibits cell invasion, enhances chemotherapeutic sensitivity in tumor cells but not in normal cells and reverts epithelial-mesenchymal transition (EMT) [5, 7]. Also, *in vivo*, DZNep inhibits tumor growth in chondrosarcoma [8], embryonal rhabdomyosarcoma [9], tongue cancer [10], head and neck squamous cell carcinoma [11], prostate cancer [12], glioblastoma [13] , cervical cancer [14] and renal cell carcinoma [15]. Beside its antiviral and antitumoral effects, DZNep might represent a novel therapeutic approach for treatment of osteoporosis [16], osteoarthritis [17], graft versus host disease [18, 19] and tissue fibrosis [20].

Despite its interest as a candidate drug in several diseases, very few reports have investigated potential side effects of DZNep. One study reported no obvious toxicity after acute administration (10 mg/kg) [21], whereas another study still investigating acute toxicity showed that DZNep causes nephrotoxicity in a dose-dependent manner in rat [22]. Additionally, at our knowledge, no research on cognitive impairments has been conducted. Herein, we investigate the chronic effects of DZNep on growth, organs, hematological parameters, activity and cognitive functions in immunocompetent NMRI mice.

## RESULTS

### Chronic DZNep treatment transiently reduced animal growth

**Figure 1:**
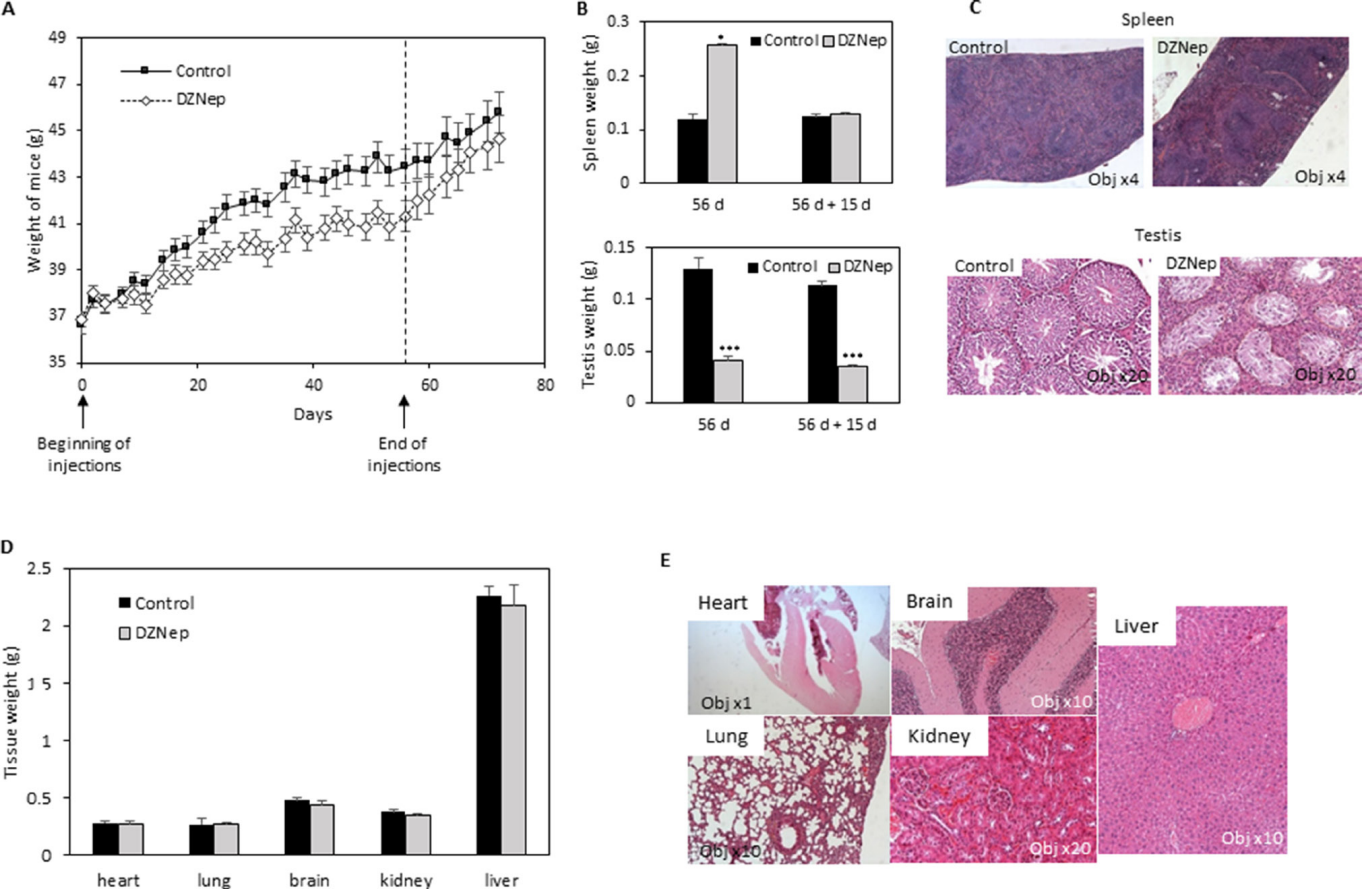
Effect of DZNep on body weight, and tissues of immunocompetent mice NMRI mice were treated for 8 weeks with DZNep (*i.p.*, 2 mg/kg/days, 3 times a week), then kept alive for two supplemental weeks without injection. Weight of mice were measured regularly during experiment (**A**). At the end of treatment (**B**, 56 d, and **D**) and 15 days later (without supplemental injections) (B, 56 d + 15 d), organs were removed and weighted. Data are expressed as means ± SEM (*n =* 3). Histological sections with HES staining were performed 15 days after the end of DZNep injections (**C** and **E**).

The chronic administration of DZNep (2 mg/kg; *i.p.*, three times per week during 8 weeks) was investigated in male NMRI mice. Animals displayed a significant decrease in body weight gain after 3 weeks of DZNep treatment, but this decrease was reversible few days after treatment arrest (Figure 1A).

### Chronic DZNep provoked a reversible splenomegaly, a persistent testis reduction and erythropoiesis

We analysed several tissues and organs after 8 weeks of treatment. Noticeably, we observed an increase in spleen weight but this splenomegaly disappears after a two-week washout period (Figure 1B). Histological analysis of spleen sections, after the wash-out, did not show difference between treated or untreated mice (Figure 1C). In another hand, we observed a decrease in testis weight and volume, both being still visible after the two week wash-out period (Figure 1B). Testis histological sections showed a hyperplasia of Leydig cells and a disappearance of spermatogonia (Figure 1C). No modification was observed in other tissues (heart, lung, brain, kidney, liver), neither in weight nor in histology (Figure 1D and 1E). Hematological analysis did not reveal difference in cell blood count, except a reduction of reticulocyte number (Table 1).

**Table 1:** Hematological parameters in NMRI mice

	Control	DZNep	*p*-value
WBC (10^9/l)	2.41 ± 0.32	1.91 ± 1.01	0.246
RBC (10^12/l)	7.61 ± 0.14	7.24 ± 1.03	0.231
HGB (g/dl)	11.86 ± 0.22	11.30 ± 1.65	0.258
MCV (fl)	54.44 ± 0.37	54.54 ± 1.94	0.875
Platelets (10^9/l)	540.79 ± 64.99	442.24 ± 104.85	0.188
PDW (fl)	6.12 ± 0.09	6.25 ± 0.88	0.623
Neutrophils (10^9/l)	0.53 ± 0.04	0.46 ± 0.16	0.321
Lymphocytes (10^9/l)	1.64 ± 0.22	1.19 ± 0.57	0.122
Monocytes (10^9/l)	0.03 ± 0.01	0.03 ± 0.03	0.943
Eosinophils (10^9/l)	0.01 ± 0.01	0.00 ± 0.01	0.504
Basophils (10^9/l)	0.00 ± 0.00	0.02 ± 0.08	0.263
**Reticulocytes (10^9/l)**	**220.91 ± 11.05**	**187.55 ± 33.76**	**0.028**
**IRF (%)**	**36.96 ± 2.46**	**30.58 ± 5.57**	**0.038**
**Ret-He (pg)**	**18.54 ± 0.15**	**17.98 ± 0.65**	**0.020**

Mice were treated with DZNep for 8 weeks. Blood analysis were done 2 weeks after the end of injections. Values represents means ± SEM. *n* = 15 for control group; *n* = 14 for DZNep group.

Abbreviations: WBC: white blood cell count, RBC: red blood cell count, HGB: hemoglobin, MCV: mean corpuscular volume, PDW: Platelet distribution Width, IRF: Immature Reticulocyte fraction; Ret-He: reticulocyte hemoglobinemia content.

**Figure 2:**
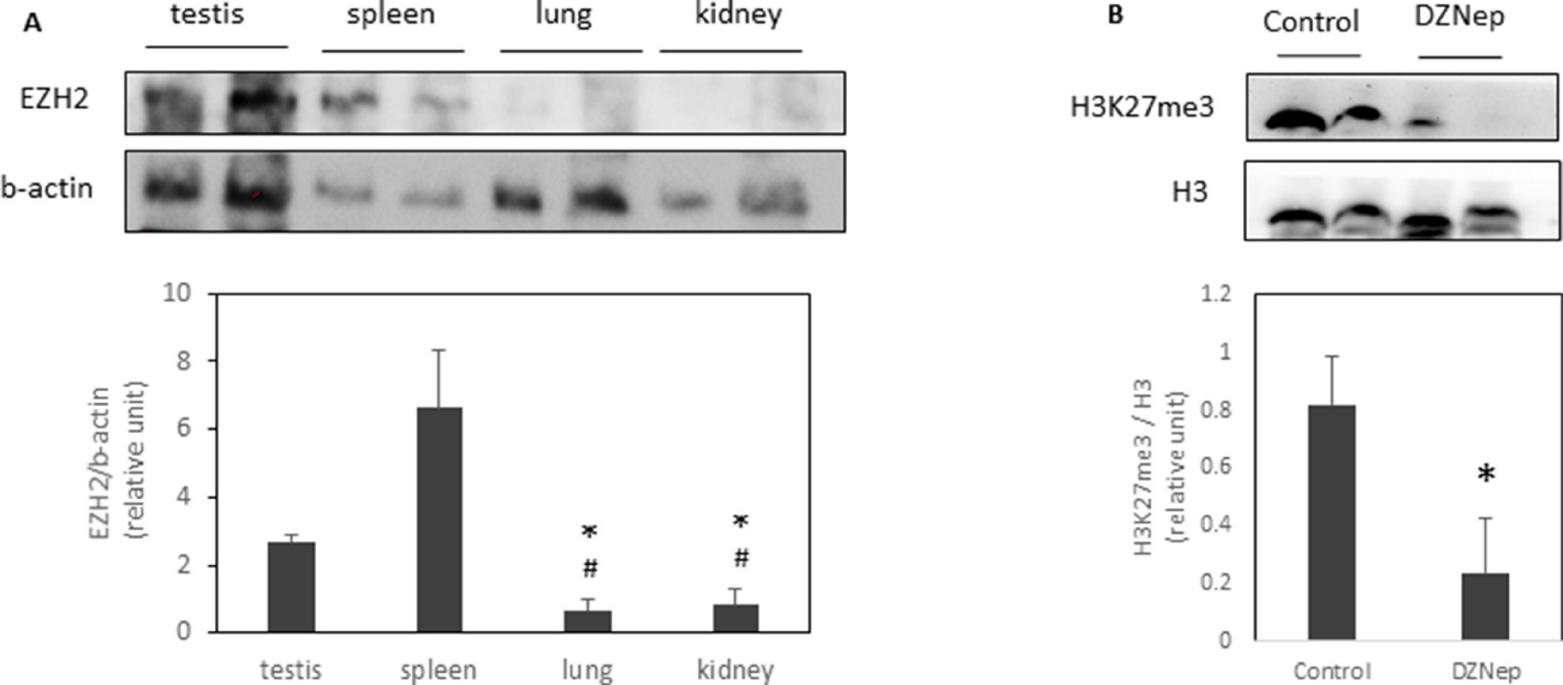
EZH2 expression and H3K27 trimethylation in several tissues EZH2 expression were analysed by western-blot in different tissues of NMRI mice (**A**). ^*^p-value < 0.05 compared to testis. ^#^*p*-value < 0.05 compared to spleen. H3K27me3 were also analysed in testis of NMRI mice treated or not with DZNep as previously (**B**). ^*^p-value < 0.05.

To understand why only some tissues were affected by DZNep, we evaluated EZH2 protein expression. We found that testis and spleen highly expressed EZH2 compared to lung or kidney (Figure 2A). Interestingly, DZNep treatment led a decrease of trimethylation of H3K27 in testis (Figure 2B).

### Chronic DZNep treatment did not affect spontaneous locomotor activity

**Figure 3:**
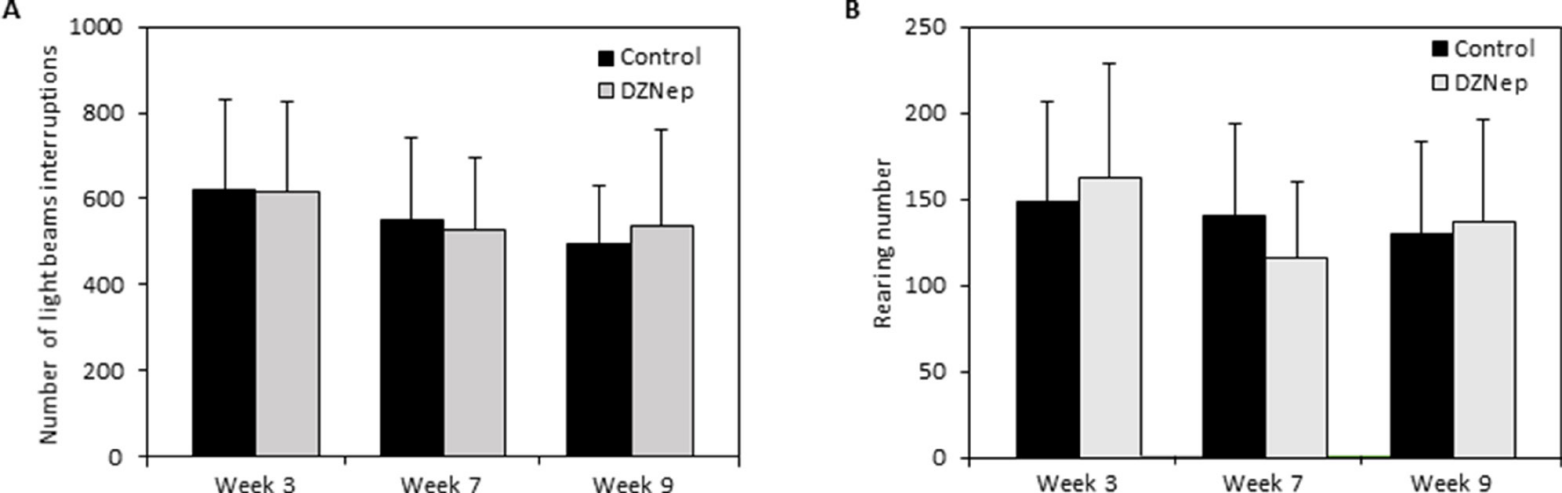
Effect of chronic DZNep injections on spontaneous locomotor activity Spontaneous locomotor activity was performed 3 and 7 weeks after the beginning of DZNep injections, and 1 week after the end of the treatment (week 9). The number of light beam interruptions (**A**) and of rearing (**B**) was evaluated in DZNep treated and control mice. Data are expressed as means ± SEM (*n =* 18).

Then, we evaluated whether DZNep affects mouse behavior. No significant difference was observed between DZNep-treated and control mice during the 30-min of activity recording, whatever the time assessed during and after the treatment. Chronic DZNep treatment had no effect neither on vertical nor on horizontal activity, as observed by the absence of difference in the light beam interruptions (Figure 3A) and number of rearing (Figure 3B).

### Chronic DZNep treatment did not modify memory performances

**Figure 4:**
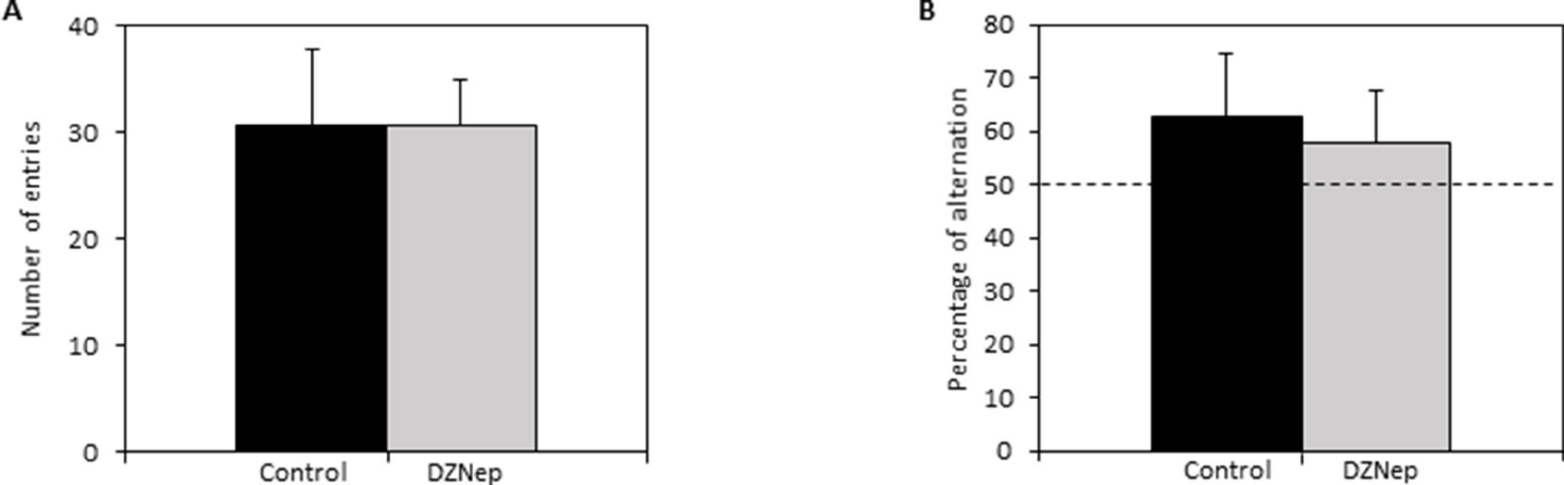
Effect of DZNep treatment on short term memory Spontaneous alternation in the Y-maze was performed at the end of the treatment (week 9; ***n* =** 15). The number of entries in each arm of Y-maze (**A**) and the percentage of spontaneous alternation in the Y-maze (**B**) were assessed in DZNep treated and untreated mice. Data are expressed as means ± SEM.

In the Y-maze, mice treated with DZNep displayed similar spontaneous alternation performances than control mice. DZNep had no effect on overall activity of mice in the maze (Figure 4A) nor on working memory performances of mice (percentage of alteration >50%; *p*-value = 0.23) (Figure 4B). The results did not reveal any deleterious effect of DZNep on working memory.

**Figure 5:**
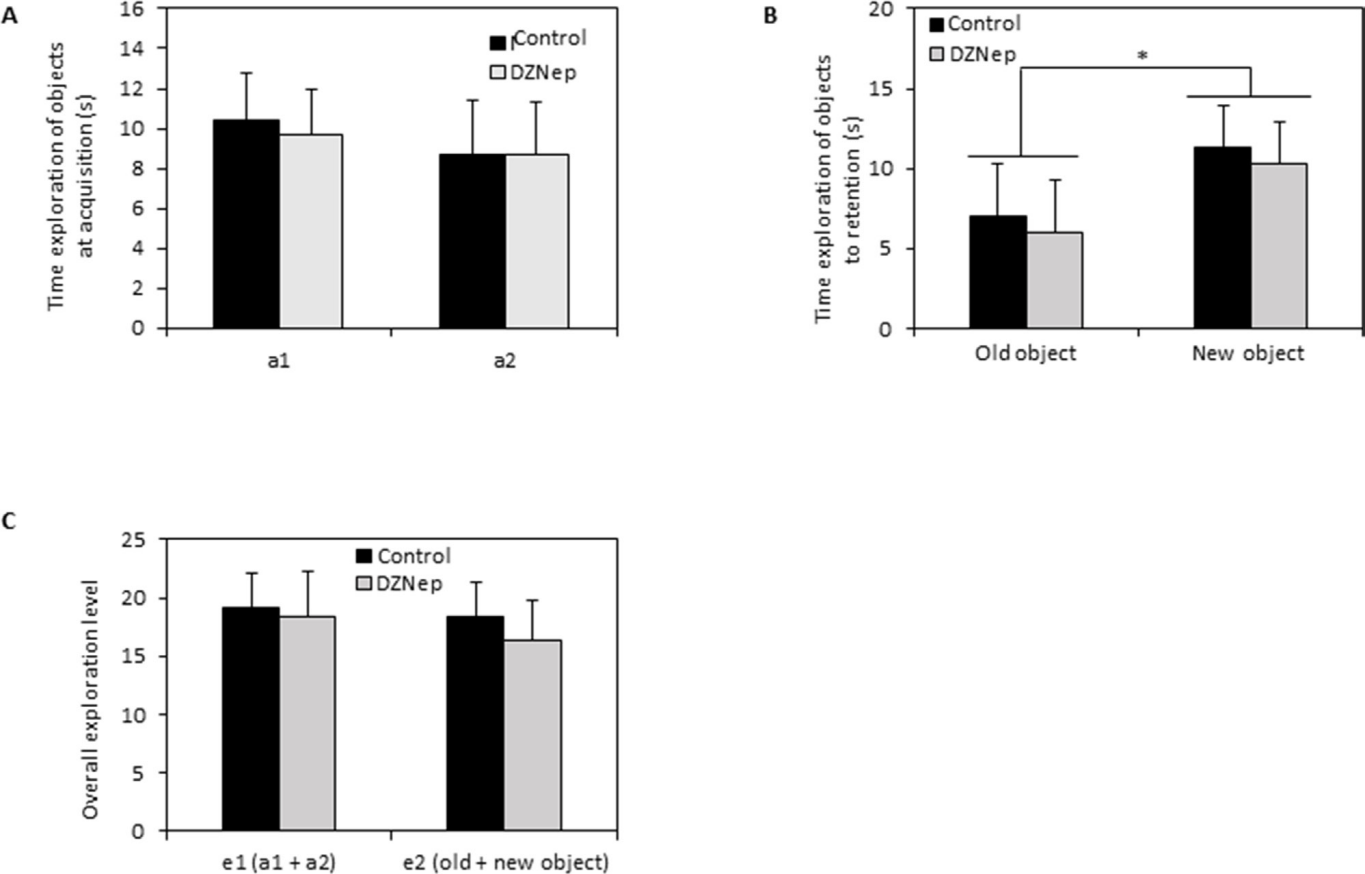
Effect of chronic DZNep treatment on long term memory Long term memory was performed by object recognition test (week 10). Time exploration of objects at acquisition (**A**) was evaluated; a1 and a2 relevant to identical object. Time exploration of objects to retention (**B**) was assessed as well as overall exploration level (**C**). Data are expressed as means ± SEM (*n =* 15).

Recognition memory was assessed by object recognition test. During the acquisition session, there was no difference in exploration time of the two identical objects between DZNep-treated and control mice (Figure 5A). During the retention session, the time spent to explore the new object was significantly increased (*p*-value = 0.0002) compared to the time spent to explore the familiar object whatever the group indicating that DZNep had no effect on recognition memory performances (Figure 5B). Overall, the level of objects exploration was not affect by chronic DZNep treatment (Figure 5C). These data suggest that DZNep had no effect on long term memory.

### Chronic DZNep treatment did not significantly influence anxiety-like behavior

**Figure 6:**
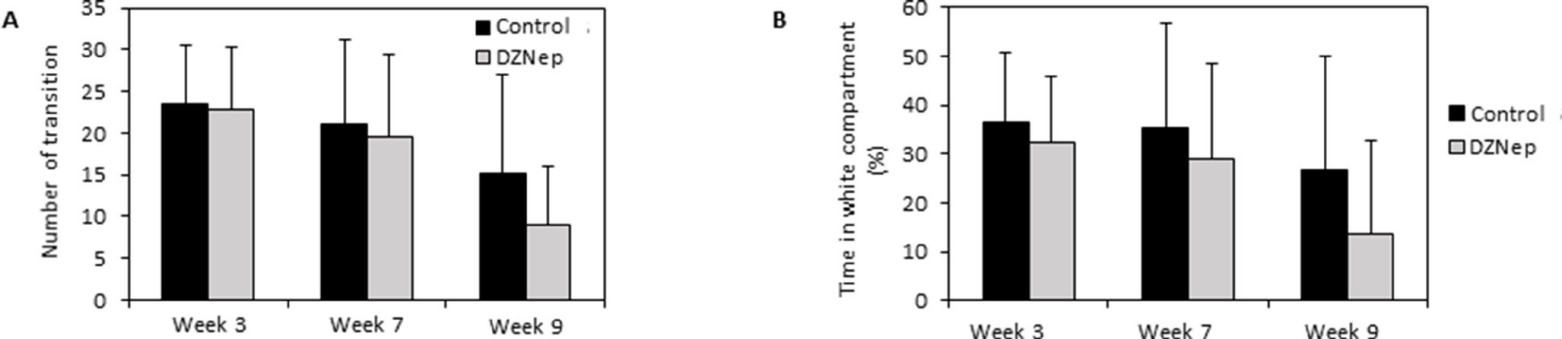
Effect of DZNep injections on anxiety-like behavior Light-dark box test was performed 3 and 7 weeks after the beginning of the treatment, and 1 week after the end of the treatment (week 9). The effect of chronic DZNep treatment on number of transition between two compartments (**A**) and percentage of time spent in the light compartment (**B**) was tested. Data are expressed as means ± SEM (*n =* 18).

Furthermore, we investigated anxiety-like behavior on the basis of preference of mice for dark and confined compared to light and open spaces. In the light-dark bow, there was no significant difference in the number of transitions between the two compartments, between treated and control mice, suggesting similar exploratory and spontaneous activity in the apparatus. However, there was a significant decrease in the number of transitions with the repetition of the test over week 3 to week 9 (theoretical average: 50%; observed average: 60%; *p*-value < 0.001) in two groups suggesting of possible habituation to the test (Figure 6A).

Furthermore, DZNep had none effect on the percentage of time spent in the light box (Figure 6B). However, one week after the end of the treatment (week 9), DZNep tended to increase the level of anxiety, as attested by a decrease in the percentage of time spent in the light box (*p*-value = 0.055) (not significant). These results suggest that chronic DZNep treatment did not significantly affect anxiety-like behavior.

### Chronic DZNep treatment did not affect depressive-like behavior

**Figure 7:**
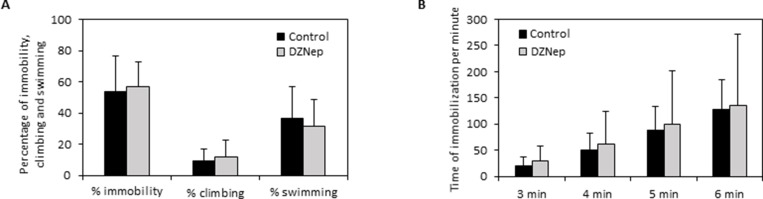
Effect of chronic DZNep treatment on depressive-like behavior Depressive-like behavior was performed by forced swimming test three weeks after the end of treatment (week 11). The percentage of immobility, climbing and swimming (**A**) and the downtime per minute (**B**) were determined in DZNep treated and control animals. Data are expressed as means ± SEM (*n* = 15).

In the forced swimming test, there was no significant difference on immobility, climbing and swimming percentages between DZNep-treated and control animals (Figure 7A). Furthermore, DZNep had no effect on time of immobilization per minute (Figure 7B). These data reveal that DZNep treatment did not affect depressive-like behavior.

## DISCUSSION

DZNep, an inhibitor of S-adenosylmethionine-dependent methyltransferase, particularly of EZH2, showed promising antitumoral effects *in vitro* and *in vivo*. Additionally, it may become a novel therapeutic approach for treatment of other diseases, such as osteoporosis, osteoarthritis or fibrosis. Herein, we tested its toxicity in immunocompetent mice. We show that chronic injection of DZNep is globally safe for animals. It has no significant effect on their behavior neither on their cognitive functions. However, it transiently reduces animal growth, and induces a reversible splenomegaly and durable testicular atrophy.

DZNep dose (2 mg/kg; 3 times a week; *i.p.*) was chosen based on previous studies that had determined doses that have an antitumoral effect *in vivo* [8, 23–25]. Herein, we show that an exposure to this treatment for 8 weeks induced a reversible decrease in body weight gain in male immunocompetent mice. However, it has been reported that female NOD/SCID immunodeficient mice tolerated well a 5 weeks DZNep treatment (5 mg/kg daily intraperitoneally), with no weight loss observed [11], and that a 4 weeks DZNep treatment (2 mg/kg; 3 times a week, *i.p.*) did not modify weight of *nude* mice (personal data). This difference could explain by the fact that it is not the same strain of mice or by implication of the immune system. However, this effect on weight gain of mice was reversible, suggesting that it is not a major trouble for mice.

Chronic DZNep injections had no effect on most biological tissues (heart, lung, brain, kidney, liver), whether in terms of weight or histology. However, they affected spleen and testis, and probably erythropoiesis. Interestingly, spleen and testis tissues highly express EZH2 [26, 27], which may explain their higher sensitivity to DZNep. This is in consistent with recent paper showing that EZH2 plays a major role in murine spermatogenesis [28], and suggests that patients treated by EZH2 inhibitors (currently in clinical trials) should have a follow-up concerning the risk of gametogenesis alteration.

In contrast, we did not observe any macroscopical or histological changes in other tissues. Notably, we could observe any abnormality in kidney after DZNep treatment while a study showed that this drug induced nephrotoxicity and renal atrophy in a dose-dependent manner in Sprague Dawley rat [22].

Additionally to these analyses, our study investigated for the first time the effects of chronic DZNep treatment on mice behavior, and especially on anxiety and depressive-like behavior, and locomotor activity as well as working and recognition memory performances. No significant difference was observed between treated and control animals although the DZNep tends to increase anxiety (not statistically significant). This was particularly interesting, since major antitumoral drugs lead to alteration of concentration, memory, executive functions, and processing speed. These data are thus encouraging for the use of DZNep in clinical trial, in particular in the context of cancerology. Indeed, this study suggests that DZNep is safer than the majority of chemotherapeutic drugs currently used in clinics. However, in the context of other pathologies (such as osteoarthritis or osteoporosis), the possible effect on anxiety, and the major alteration of testis histology are major concerns, and make necessary either to reduce dose of treatment, or to target the altered tissues (for instance joint for osteoarthritis, or bone for osteoporosis).

In conclusion, the present study is the first which focuses on chronic effects of DZNep in immunocompetent mice with behavioral tests, and organ and hematological analysis. Although DZNep has not yet been tested in clinical trials, we show in mice that DZNep has no effect on behavior and induces side effects irreversible limited only on testis which is unfortunately found in most chemotherapy treatment.

## MATERIALS AND METHODS

### Animals

Animal experimental procedures were performed according to local legislation, and procedures were approved by ethics committee (Comité d’Ethique Normandie en Matière d’Expérimentation Animale, agreement #03968.01). The animals were provided and kept in the animal facility (Centre Universitaire de Ressources Biologiques, Caen, France) under controlled temperature and light conditions (temperature 23 ± 2° C, 12 h reversed light-dark cycle). Animals had *ad libitum* access to food and water. Each animal was humanely handled throughout the experiment in accordance with internationally accepted ethical principles for laboratory animal use and care, and all efforts were made to minimize animal suffering.

### Animal treatment

**Figure 8:**
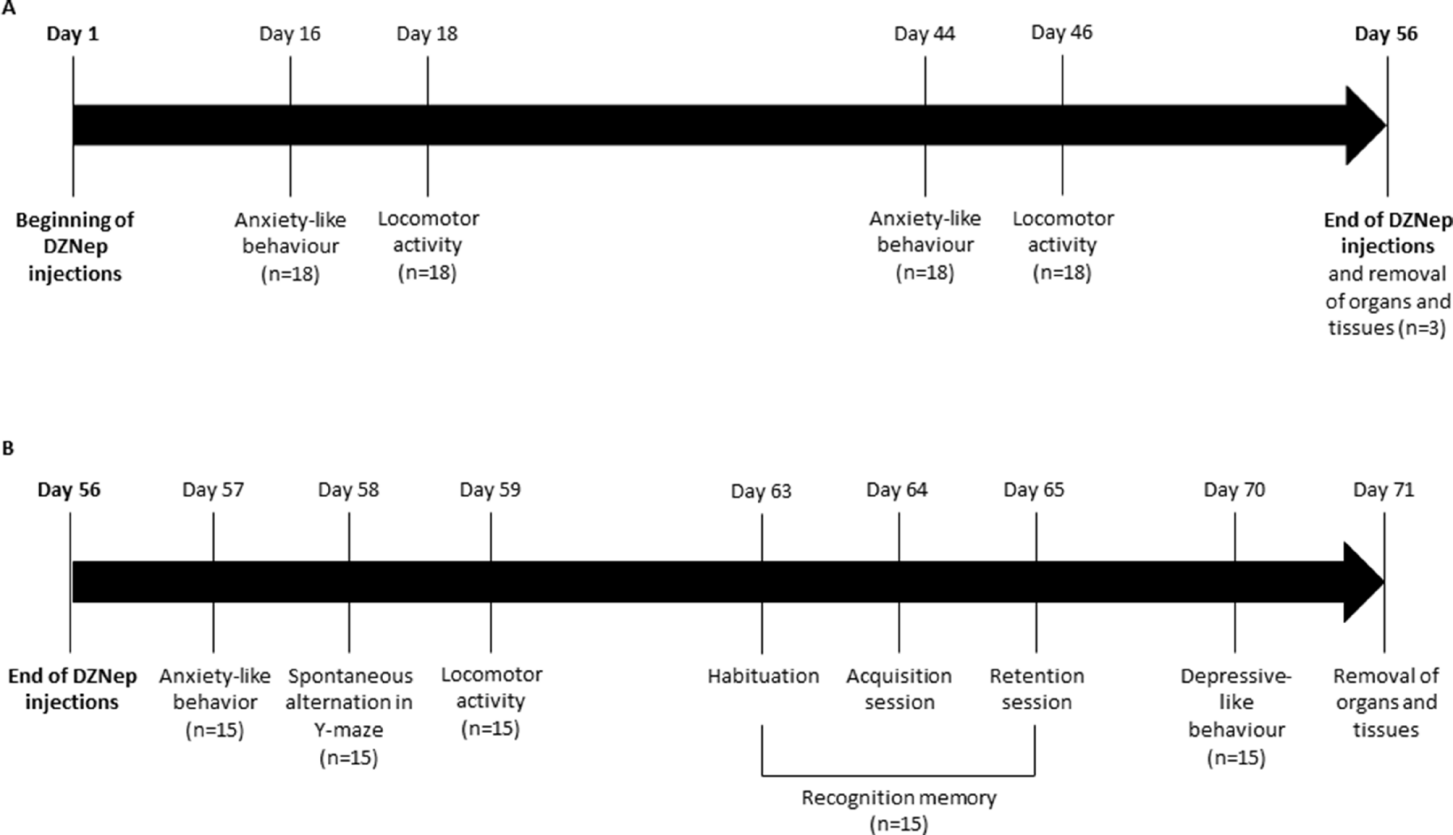
Experimental design NMRI mice were treated for 8 weeks (56 days) with DZNep (*i.p.*, 2 mg/kg, 3 times per week) (*n =* 18/group) (**A**). Anxiety-like behavior and locomotor activity were assessed at weeks 2 and 6. At week 8 (*e.g.* at the end of treatment), organs and tissues were removed for three mice/group. For other animals (*n =* 15/group), mice were kept alive during two additional weeks without injections (**B**). Anxiety-like behavior, spontaneous alternation in Y-maze and locomotor activity were performed one, two and three days after the end of treatments (day 57, 58 and 59) respectively. Recognition memory was assessed one week after the end of treatment, during 3 days with different sessions (day 63: habituation session, day 64: acquisition session and day 65: retention session). Finally, depressive-like behavior was performed two weeks after the end of treatment (day 70). Organs and tissues were removed the next day.

The experiment was performed on male NMRI mice (8 weeks old). Two groups of mice (*n =* 18/group) receiving either DZNep (2 mg/kg; intraperitoneal injection; three times per week during 8 weeks; dissolved in NaCl 0.9%; provided by Tocris, Lille, France) or vehicle (NaCl 0.9%) were constituted. A follow-up of body weight was performed during all experiment and behavior experiments were performed. At the end of the treatment (8 weeks), 6 animals (3/group) were euthanized by cervical dislocation and several organs (spleen, testis, lung, heart, brain, kidney and liver) were collected for analysis. During washout period (2 weeks after the end of the treatment), behavior experiments were continued and at the end of the 10 weeks (*i.e.* 8 weeks of treatment followed by 2 weeks of washout), animals were anesthetized (isoflurane 3%, in a mixture ½ ^O^_2_^/N^_2_^O) and^ intracardiac blood samples ^and^ several organs (spleen, testis, lung, heart, brain, kidney, liver) were collected for hematological and histopathological analysis (Figure 8).

### Protein extraction and western-blot

Tissues were incubated into RIPA lysis buffer (Tris-HCl 50 mM pH 7,5; IGEPAL 1%; NaCl 150 mM; EGTA 1 mM; NaF 1 mM) supplemented with phosphatase (NA_3_VO_4_ 10 µl/ml) and protease inhibitors (leupeptin 1 µL/mL, aprotinin 1 µL/mL, pepstatin 1 µL/mL and phenylmethylsulfonyl fluoride 4 µL/mL). Protein extracts were resolved by SDS-PAGE, and transferred to polyvinylidene difluoride (PVDF) membranes (Bio-Rad). After probing with primary antibodies (EZH2: #5246 Cell Signaling; H3K27me3: #9733 Cell Signaling; H3: #4499 Cell Signaling; β-actin: sc-47778, Santa Cruz Biotechnology), the membranes were incubated with horseradish peroxidase-conjugated secondary antibodies, and signals visualized by Western Lightning^®^ Plus-ECL (Perkin Elmer).

### Histology tissues

After resection, organs (testis, spleen, brain, lung, heart, kidney and liver) were weighed and fixed in 20% formalin during 48 hours and embedded in paraffin. The sections were stained with hematoxylin, eosin and saffron, and the histological aspect of the different organs was analysed by standard light microscopy.

### Hematological analysis

Samples of 3.2% citrated blood were collected after cardiac punctures. Blood samples were analysed with the multiple parameters automated hematology analyser XN-9000 (Sysmex France, Villepinte, France) within 6 hours after blood draw. Counts of platelet count, hemoglobin count, red and white cells count, neutrophils count, lymphocytes, monocytes, eosinophil, basophils and reticulocytes were determined using the impedance channel (Hématologie biologique, CHU de Caen, Caen, France).

### Locomotor activity

Spontaneous activity was recorded in actimeter units (IMETRONIC^®^, France) as described previously [29]. The number of light beams interruptions (two level of beams for horizontal and vertical activity) was recorded during a single session of 30 min and used as an index of spontaneous locomotor activity. Locomotor activity was assessed 18 and 46 days after the beginning of the treatment and 3 days after the end of the treatment (Figure 8).

### Spontaneous alternation in Y-maze

Spatial working memory performances were assessed by recording spontaneous alternation behavior in a single-session Y-maze test [29]. Experiment was assessed at the end of the treatment (week 8) (Figure 8). The maze consisted of three equally spaced arms (22 cm long, 6.5 cm wide, walls of 10 cm high) made of black-painted wood. The mouse was placed at the end of one arm and allowed to explore the maze freely during a 5 min session. The number and the sequence of arm entries and rearing were recorded by the observer. An arm entry was scored when all four feet crossed into the arm. Alternation behavior was defined as consecutive entries into all three arms. The percentage of alternation was calculated as a memory index by the (number of alternation/maximal theoretical number of alternation) × 100.

### Recognition memory

Object recognition test was performed in 2 sessions separated by a 1 h inter-trial interval (ITI). Experiment was performed in an open field (33 × 33 × 20 cm, wood painted black), with four duplicates of two different objects available (Lego^®^ vs Falcon^®^) [30]. Recognition memory was assessed one week after the end of the treatment (week 9) (Figure 8). The first day, mice was placed alone in the open field during 5 min for habituation of the box. The second day, each mouse was placed in the open field during 5 min with an unknown object different from those used during the test. Finally, on the third day, two experimental trials (6 min each or 20 s accumulated for exploration of the 2 objects, separated by a 1 h ITI) were performed. During the first trial (acquisition session), the mouse was allowed to explore two identical objects (a1 and a2) before being placed back in its home cage. For the second trial (retention session), two different objects were used — an identical copy of the familiar object (a3) and a novel one (b). Between animals, the relative position of the two objects was alternated randomly. The objects and the open field were cleaned with diluted 70% ethanol between each trial. Exploration time was defined as the time spent with the nose at a distance less than 2 cm from the object, directed to the objet, or touching the object with forelimbs. Exploration times were manually recorded (stopwatches) by an experimenter blind to the treatment group, located one meter from the apparatus and by indirect observation of the animal in a mirror. A discrimination index, on which the analysis were performed, was then calculated from the exploration times: [novel object−familiar object]/[novel object+familiar object].

### Anxiety-like behavior

Anxiety-like behavior was assessed in the light-dark box (LETICA LE 810, Bioseb^®^, France) on the basis of the innate preference of mice for dark and confined compared to light and open spaces. Anxiety-like behavior was performed 16 and 44 days after the beginning of the treatment and the day after the end of the treatment (Figure 8). The mouse was placed in the center of the illuminated white compartment (28 × 27 × 27 cm, 900 lux) and left to freely explore the apparatus for 5 min. The latency to enter the dark compartment (17 × 27 × 27 cm, 100 lux, red light, 120 s maximum), the number of transitions between the compartments and the time spent in each compartment were collected. The percentage of time spent in the illuminated compartment was used as an index of anxiety-like behavior.

### Depressive-like behavior

Forced swimming test is based on the immobile posture adopted by mice when exposed to an inescapable stressor and is used to detect potential antidepressant effects of drugs. Depressive-like behavior was assessed two weeks after the end of the treatment (week 10) (Figure 8). The apparatus consisted of a transparent glass cylinder (30 cm height, 15 cm diameter) containing water (20 cm deep) at 25 ± 1° C. The mouse was placed in the water and the immobility time, the time of swimming and the time of climbing were collected during the last 4 min of the 6 min testing period as already described [31]. The latency was also recorded.

### Statistical analysis

All data were expressed as mean ± SEM. Statistical significance was determined with Student’s test for hematological analysis. According to the behavioral test, a one or two-way ANOVA with repeated measurements was performed. When necessary, a Fisher’s PLSD test, as posthoc test, was performed. For spontaneous alternation in Y-maze, Univariate *T*-test compared to 50% of alternation was performed. *P*-values ≤ 0.05 were considered as statistically significant.
